# Hexaaqua­(5,7-dihydr­oxy-4-oxo-2-phenyl-4*H*-chromene-8-sulfonato)calcium(II) 5,7-dihydr­oxy-4-oxo-2-phenyl-4*H*-chromene-8-sulfonate trihydrate

**DOI:** 10.1107/S1600536808037586

**Published:** 2008-11-20

**Authors:** Bin Liu, Bo-Lun Yang

**Affiliations:** a School of Energy and Power Engineering, Xi’an Jiaotong University, Xi’an 710049, People’s Republic of China; b Department of Chemistry, Xianyang Normal University, Xianyang 712000, People’s Republic of China

## Abstract

In the title compound, [Ca(C_15_H_9_O_7_S)(H_2_O)_6_](C_15_H_9_O_7_S)·3H_2_O, the Ca centre has a distorted deca­hedral geometry, coordinated by six O atoms from water mol­ecules and one sulfonate O atom. The crystal structure is stabilized by aromatic π–π inter­actions, with centroid–centroid distances of 3.765 (5) and 3.896 (5) Å between the phenyl ring and the benzene ring of the chromene unit of neighbouring mol­ecules. In addition, the stacked mol­ecules exhibit inter- and intra­molecular O—H⋯O hydrogen bonds, including the uncoordinated water mol­ecules.

## Related literature

For biological activity, see: Chan *et al.* (2000 ▶); Hiroyuki *et al.* (1996 ▶); Jiang *et al.* (2001 ▶); Lee *et al.* (1999 ▶); Shin *et al.* (1999 ▶); Zanoli *et al.* (2000 ▶). For related structures, see: Cote & Shimizu (2003 ▶); Li & Zhang (2008 ▶); Morin *et al.* (2000 ▶); Pusz *et al.* (2001 ▶); Zhang *et al.* (2004 ▶, 2006*a* ▶,*b* ▶).
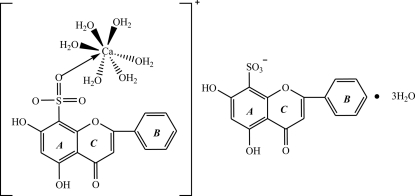


## Experimental

### 

#### Crystal data

[Ca(C_15_H_9_O_7_S)(H_2_O)_6_](C_15_H_9_O_7_S)·3H_2_O
*M* *_r_* = 868.79
Triclinic, 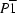

*a* = 11.360 (2) Å
*b* = 12.390 (1) Å
*c* = 13.975 (2) Å
α = 95.136 (2)°
β = 102.167 (3)°
γ = 107.423 (2)°
*V* = 1809.9 (4) Å^3^
*Z* = 2
Mo *K*α radiation
μ = 0.38 mm^−1^
*T* = 296 (2) K
0.36 × 0.23 × 0.14 mm

#### Data collection

Bruker SMART CCD area-detector diffractometer
Absorption correction: multi-scan (*SADABS*; Bruker, 1999 ▶) *T*_min_ = 0.874, *T*_max_ = 0.947
9179 measured reflections
6299 independent reflections
4416 reflections with *I* > 2σ(*I*)
*R*_int_ = 0.026

#### Refinement

*R*[*F* ^2^ > 2σ(*F* ^2^)] = 0.055
*wR*(*F* ^2^) = 0.167
*S* = 1.04
6299 reflections
558 parameters
16 restraints
H atoms treated by a mixture of independent and constrained refinement
Δρ_max_ = 0.40 e Å^−3^
Δρ_min_ = −0.40 e Å^−3^

### 

Data collection: *SMART* (Bruker, 1999 ▶); cell refinement: *SAINT-Plus* (Bruker, 1999 ▶); data reduction: *SAINT-Plus*; program(s) used to solve structure: *SHELXS97* (Sheldrick, 2008 ▶); program(s) used to refine structure: *SHELXL97* (Sheldrick, 2008 ▶); molecular graphics: *SHELXTL* (Sheldrick, 2008 ▶); software used to prepare material for publication: *SHELXTL*.

## Supplementary Material

Crystal structure: contains datablocks I, global. DOI: 10.1107/S1600536808037586/lx2072sup1.cif

Structure factors: contains datablocks I. DOI: 10.1107/S1600536808037586/lx2072Isup2.hkl

Additional supplementary materials:  crystallographic information; 3D view; checkCIF report

## Figures and Tables

**Table 1:** Hydrogen-bond geometry (Å, °)

*D*—H⋯*A*	*D*—H	H⋯*A*	*D*⋯*A*	*D*—H⋯*A*
O3—H3⋯O2	0.82	1.85	2.576 (4)	148
O4—H4⋯O5	0.82	1.82	2.579 (4)	153
O8—H8*B*⋯O17^i^	0.82 (4)	2.20 (3)	2.934 (4)	149 (4)
O8—H8*A*⋯O20^ii^	0.82 (3)	1.97 (3)	2.790 (4)	176 (5)
O9—H9*B*⋯O5	0.82 (4)	1.99 (4)	2.780 (4)	163 (5)
O9—H9*A*⋯O23^iii^	0.83 (3)	1.92 (3)	2.743 (4)	175 (5)
O10—H10*B*⋯O20^ii^	0.82 (3)	2.13 (2)	2.874 (4)	151 (5)
O10—H10*A*⋯O22	0.82 (3)	1.90 (3)	2.715 (4)	171 (5)
O11—H11*B*⋯O6^iii^	0.82 (3)	2.36 (3)	3.024 (4)	139 (4)
O11—H11*A*⋯O19	0.82 (4)	2.19 (4)	3.003 (4)	175 (5)
O12—H12*B*⋯O15^iv^	0.81 (3)	1.98 (3)	2.771 (4)	165 (5)
O12—H12*A*⋯O21	0.82 (4)	1.88 (4)	2.695 (5)	177 (4)
O13—H13*B*⋯O3^v^	0.82 (4)	2.05 (4)	2.867 (4)	174 (5)
O13—H13*A*⋯O23	0.82 (3)	2.00 (3)	2.819 (4)	173 (5)
O16—H16⋯O15	0.82	1.83	2.567 (4)	148
O17—H17⋯O18	0.82	1.80	2.556 (4)	153
O21—H21*B*⋯O18	0.82 (3)	2.05 (4)	2.870 (4)	176 (5)
O21—H21*A*⋯O22^ii^	0.82 (3)	2.01 (3)	2.826 (5)	173 (5)
O23—H23*A*⋯O2^vi^	0.82 (4)	2.00 (4)	2.816 (4)	171 (5)
O23—H23*B*⋯O6	0.82 (4)	1.95 (4)	2.755 (4)	168 (5)
C12—H12⋯O11^i^	0.93	2.55	3.462 (5)	168
C20—H20⋯O8^vii^	0.93	2.52	3.410 (5)	161

Symmetry codes: (i) 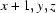; (ii) 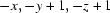; (iii) 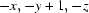; (iv) 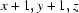; (v) 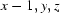; (vi) 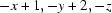; (vii) 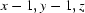.

**Fig. 1.**
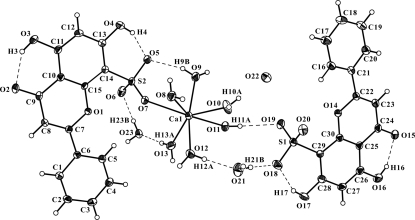
The molecular structure of the title compound, showing displacement ellipsoids drawn at the 30% probability level. Hydrogen bonds are illustrated as dashed lines.

**Fig. 2.**
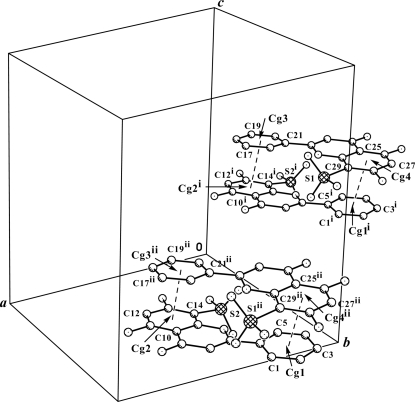
π–π interactions (dotted lines) in the title compound. Cg denotes the ring centroid. [Symmetry codes: (i) x - 1, y - 1, z; (ii) x + 1, y + 1, z.]

## supplementary crystallographic information

### Comment

Chrysin (5,7-dihydroxyflavone), a naturally wide distributed flavonoid, has many different biological activities such as anti-oxidant (Chan *et al.*, 2000), anti-virus (Lee *et al.*, 1999), anti-diabetogenic activity (Shin *et al.*, 1999), anti-anxiolytic effect (Zanoli *et al.*, 2000). Sulfonates belong to an important class of organic compounds, particularly flavonoidsulfonates have many different biological activities (Hiroyuki *et al.*, 1996; Jiang *et al.*, 2001). Previously, two chrysinsulfonate derivatives have been prepared. Zhang *et al.* (2004, 2006*a*, 2006*b*) have synthesized chrysin-6-sulfonate and its derivates, such as [Ba(C_15_H_9_O_7_S)_2_]_n_, [Zn(C_15_H_8_O_7_S)(DMSO)]_2_.H_2_O and [{Ca(C_15_H_8_O_7_S)(H_2_O)(DMSO)}_3_ {Ca(C_15_H_8_O_7_S)(DMSO)_2_}].4DMSO. Pusz *et al.* (2001) have reported chrysin-4'-sulfonate and its Ti^4+^, Mn^2+^ and Fe^3+^ complexes. On the other hand, the weak coordination nature of SO3^-^ makes its coordination mode very flexible and sensitive to the chemical environment (Cote & Shimizu, 2003). Here we report the crystal structure of the title compound (Fig. 1).

As shown in Fig. 1, the Ca atom is seven-coordinated by the six O atoms from water molecules and the one sulfonate O atom. The Ca—O bond lengths are in agreement with the corresponding values in [Ca(C_16_H_12_O_4_)(H_2_O)_6_]. H_2_O (Morin *et al.*, 2000). The flavone skeleton is essentially planar, the bond lengths and angles are similar to those reported for other flavonesulfonates, [Co(H_2_O)_6_](C_16_H_11_O_7_S)_2_.4H_2_O (Li & Zhang, 2008). The molecular packing (Fig. 2) is stabilized by two different aromatic π–π interactions within each stack of molecule; one between the phenyl ring (*Cg*1) and the benzene ring (*Cg*4^ii^) of the adjacent molecules {distance; 3.765 (5) Å}, and the other between the benzene ring (*Cg*2) and the phenyl ring (*Cg*3^ii^) of the neighbouring molecules {distance; 3.896 (5) Å} (Fig. 2; *Cg*1, *Cg*2, *Cg*3 and *Cg*4 are the centroids of the C1–C6 phenyl, the C10–C15 benzene, the C16–C21 phenyl and the C25–C30 benzene rings, respectively, symmetry code as in Fig. 2). Additionally, the crystal structure exhibits numerous inter- and intramolecular O—H···O hydrogen bonds (Fig. 1 & Hydrogen-bond geometry).

### Experimental

5,7-Dihydroxyflavone (chrysin, 1.0 g, 3.9 mmol) was added slowly to concentrated sulfuric acid (6 ml) with stirring. The reaction was maintained at room temperature for 12 h. Then, it was poured into NaCl saturated aqueous solution (50 ml) and a yellow precipitate appeared. After 5 h, the precipitate was filtered and washed with NaCl saturated aqueous solution until the pH value of the filtrate was 7. It was dissolved in water (50 ml), and mixed with saturated CaCl_2_ solution (10 ml). (I) was obtained after 24 h. It was recrystallized from an ethanol-water (1:1 *v/v*) solution. Colorless sheet-shaped crystals suitable for X-ray analysis were obtained by slow evaporation of the solvent for about 3 d at room temperature (yield 78%).

### Refinement

H atoms were positioned geometrically and refined as riding atoms, with C—H = 0.93 (aromatic) and O—H = 0.82 (phenolic) Å, *U*_iso_(H) = 1.2Ueq(C) and *U*_iso_(H) = 1.5Ueq(O). H atoms of the water molecules were found in difference maps and positionally refined with constraints of *U*_iso_(H) = 1.5*U*_eq_(O). The reasonable position of H atoms in O22 were not obtained because of short inter distance with O19.

### Crystal data

**Table:**

[Ca(C_15_H_9_O_7_S)(H_2_O)_6_](C_15_H_9_O_7_S)·3H_2_O	*Z* = 2
*M**_r_* = 868.79	*F*_000_ = 904
Triclinic, *P*1	*D*_x_ = 1.594 Mg m^−^^3^
Hall symbol: -P 1	Mo *K*α radiation λ = 0.71073 Å
*a* = 11.360 (2) Å	Cell parameters from 2128 reflections
*b* = 12.390 (1) Å	θ = 2.5–23.9º
*c* = 13.975 (2) Å	µ = 0.38 mm^−^^1^
α = 95.136 (2)º	*T* = 296 (2) K
β = 102.167 (3)º	Sheet, colourless
γ = 107.423 (2)º	0.36 × 0.23 × 0.14 mm
*V* = 1809.9 (4) Å^3^	

### Data collection

**Table:**

Bruker SMART CCD area-detector diffractometer	6299 independent reflections
Radiation source: fine-focus sealed tube	4416 reflections with *I* > 2σ(*I*)
Monochromator: graphite	*R*_int_ = 0.026
Detector resolution: 10.0 pixels mm^-1^	θ_max_ = 25.1º
*T* = 296(2) K	θ_min_ = 1.5º
φ and ω scans	*h* = −9→13
Absorption correction: multi-scan(SADABS; Bruker, 1999)	*k* = −14→14
*T*_min_ = 0.874, *T*_max_ = 0.947	*l* = −16→16
9179 measured reflections	

### Refinement

**Table:**

Refinement on *F*^2^	Secondary atom site location: difference Fourier map
Least-squares matrix: full	Hydrogen site location: inferred from neighbouring sites
*R*[*F*^2^ > 2σ(*F*^2^)] = 0.055	H atoms treated by a mixture of independent and constrained refinement
*wR*(*F*^2^) = 0.167	*w* = 1/[σ^2^(*F*_o_^2^) + (0.0956*P*)^2^] where *P* = (*F*_o_^2^ + 2*F*_c_^2^)/3
*S* = 1.04	(Δ/σ)_max_ < 0.001
6299 reflections	Δρ_max_ = 0.40 e Å^−^^3^
558 parameters	Δρ_min_ = −0.40 e Å^−^^3^
16 restraints	Extinction correction: none
Primary atom site location: structure-invariant direct methods	

### Special details

**Table:**

Geometry. All esds (except the esd in the dihedral angle between two l.s. planes) are estimated using the full covariance matrix. The cell esds are taken into account individually in the estimation of esds in distances, angles and torsion angles; correlations between esds in cell parameters are only used when they are defined by crystal symmetry. An approximate (isotropic) treatment of cell esds is used for estimating esds involving l.s. planes.
Refinement. Refinement of F^2^ against ALL reflections. The weighted R-factor wR and goodness of fit S are based on F^2^, conventional R-factors R are based on F, with F set to zero for negative F^2^. The threshold expression of F^2^ > 2sigma(F^2^) is used only for calculating R-factors(gt) etc. and is not relevant to the choice of reflections for refinement. R-factors based on F^2^ are statistically about twice as large as those based on F, and R- factors based on ALL data will be even larger.

### Fractional atomic coordinates and isotropic or equivalent isotropic displacement parameters (Å^2^)

**Table:**

	*x*	*y*	*z*	*U*_iso_*/*U*_eq_	
Ca1	0.10081 (6)	0.62071 (6)	0.23114 (5)	0.0393 (2)	
S2	0.33030 (8)	0.65555 (7)	0.07174 (7)	0.0427 (3)	
O1	0.46932 (19)	0.90038 (18)	0.11438 (16)	0.0358 (5)	
O2	0.8501 (2)	1.0701 (2)	0.17925 (19)	0.0492 (6)	
O3	0.8907 (2)	0.8768 (2)	0.1856 (2)	0.0551 (7)	
H3	0.9073	0.9455	0.1833	0.083*	
O4	0.5425 (3)	0.5415 (2)	0.1183 (3)	0.0636 (8)	
H4	0.4644	0.5167	0.1030	0.095*	
O5	0.3082 (3)	0.5319 (2)	0.0591 (2)	0.0645 (8)	
O6	0.2916 (2)	0.6914 (2)	−0.02215 (19)	0.0582 (7)	
O7	0.2732 (2)	0.6922 (2)	0.14628 (19)	0.0489 (6)	
O8	0.3056 (3)	0.6613 (3)	0.3527 (2)	0.0547 (7)	
H8A	0.289 (4)	0.653 (4)	0.4063 (18)	0.080*	
H8B	0.332 (4)	0.612 (3)	0.331 (3)	0.080*	
O9	0.1102 (3)	0.4520 (2)	0.1496 (3)	0.0698 (9)	
H9A	0.053 (3)	0.390 (2)	0.128 (3)	0.080*	
H9B	0.170 (3)	0.462 (4)	0.124 (3)	0.080*	
O10	0.0505 (3)	0.5170 (3)	0.3609 (2)	0.0716 (9)	
H10A	0.011 (4)	0.4479 (12)	0.345 (3)	0.080*	
H10B	0.088 (4)	0.534 (4)	0.4197 (12)	0.080*	
O11	−0.1277 (3)	0.5121 (2)	0.1799 (2)	0.0533 (7)	
H11B	−0.169 (4)	0.487 (4)	0.1221 (13)	0.080*	
H11A	−0.171 (4)	0.473 (3)	0.212 (3)	0.080*	
O12	0.0807 (3)	0.7627 (2)	0.3400 (2)	0.0597 (8)	
H12A	0.039 (4)	0.746 (4)	0.381 (2)	0.065 (15)*	
H12B	0.096 (4)	0.8316 (11)	0.343 (3)	0.080*	
O13	0.0262 (3)	0.7350 (3)	0.1202 (2)	0.0633 (8)	
H13B	−0.009 (4)	0.780 (3)	0.137 (3)	0.080*	
H13A	0.036 (5)	0.742 (4)	0.0644 (16)	0.080*	
C1	0.4349 (3)	1.1836 (3)	0.1122 (3)	0.0416 (8)	
H1	0.5183	1.2318	0.1219	0.050*	
C2	0.3361 (4)	1.2284 (3)	0.0989 (3)	0.0489 (9)	
H2	0.3534	1.3068	0.0996	0.059*	
C3	0.2126 (4)	1.1585 (3)	0.0846 (3)	0.0528 (10)	
H3A	0.1466	1.1894	0.0751	0.063*	
C4	0.1863 (4)	1.0421 (3)	0.0843 (3)	0.0533 (10)	
H4A	0.1026	0.9949	0.0752	0.064*	
C5	0.2837 (3)	0.9957 (3)	0.0974 (3)	0.0438 (9)	
H5	0.2654	0.9173	0.0973	0.053*	
C6	0.4093 (3)	1.0654 (3)	0.1110 (2)	0.0341 (7)	
C7	0.5135 (3)	1.0167 (3)	0.1252 (2)	0.0329 (7)	
C8	0.6398 (3)	1.0755 (3)	0.1468 (3)	0.0383 (8)	
H8	0.6672	1.1550	0.1527	0.046*	
C9	0.7315 (3)	1.0169 (3)	0.1605 (2)	0.0373 (8)	
C10	0.6825 (3)	0.8946 (3)	0.1508 (2)	0.0364 (8)	
C11	0.7630 (3)	0.8262 (3)	0.1641 (3)	0.0408 (8)	
C12	0.7130 (3)	0.7104 (3)	0.1546 (3)	0.0485 (9)	
H12	0.7671	0.6669	0.1655	0.058*	
C13	0.5817 (3)	0.6561 (3)	0.1287 (3)	0.0435 (8)	
C14	0.4975 (3)	0.7200 (3)	0.1131 (3)	0.0373 (8)	
C15	0.5518 (3)	0.8389 (3)	0.1270 (2)	0.0334 (7)	
S1	−0.31929 (8)	0.40326 (7)	0.37480 (7)	0.0416 (2)	
O14	−0.4556 (2)	0.16187 (18)	0.37202 (17)	0.0391 (6)	
O15	−0.8338 (2)	−0.0055 (2)	0.3292 (2)	0.0579 (7)	
O16	−0.8755 (2)	0.1864 (2)	0.3212 (3)	0.0693 (9)	
H16	−0.8920	0.1170	0.3196	0.104*	
O17	−0.5301 (3)	0.5183 (2)	0.3526 (3)	0.0708 (9)	
H17	−0.4520	0.5427	0.3656	0.106*	
O18	−0.2978 (3)	0.5258 (2)	0.3808 (3)	0.0689 (9)	
O19	−0.2916 (2)	0.3558 (2)	0.28751 (19)	0.0521 (7)	
O20	−0.2531 (2)	0.3747 (2)	0.46429 (19)	0.0556 (7)	
C16	−0.2675 (3)	0.0700 (3)	0.4116 (3)	0.0474 (9)	
H16A	−0.2490	0.1491	0.4187	0.057*	
C17	−0.1700 (4)	0.0242 (4)	0.4252 (3)	0.0601 (11)	
H17A	−0.0859	0.0725	0.4406	0.072*	
C18	−0.1959 (4)	−0.0926 (4)	0.4162 (3)	0.0614 (11)	
H18	−0.1297	−0.1232	0.4251	0.074*	
C19	−0.3201 (4)	−0.1637 (3)	0.3939 (3)	0.0613 (11)	
H19	−0.3379	−0.2425	0.3893	0.074*	
C20	−0.4183 (4)	−0.1186 (3)	0.3784 (3)	0.0543 (10)	
H20	−0.5022	−0.1675	0.3619	0.065*	
C21	−0.3936 (3)	−0.0018 (3)	0.3871 (2)	0.0396 (8)	
C22	−0.4987 (3)	0.0466 (3)	0.3714 (2)	0.0392 (8)	
C23	−0.6230 (4)	−0.0108 (3)	0.3576 (3)	0.0460 (9)	
H23	−0.6490	−0.0896	0.3570	0.055*	
C24	−0.7170 (3)	0.0460 (3)	0.3437 (3)	0.0440 (9)	
C25	−0.6679 (3)	0.1687 (3)	0.3477 (3)	0.0398 (8)	
C26	−0.7489 (3)	0.2363 (3)	0.3370 (3)	0.0486 (9)	
C27	−0.6996 (3)	0.3518 (3)	0.3409 (3)	0.0537 (10)	
H27	−0.7534	0.3958	0.3355	0.064*	
C28	−0.5694 (4)	0.4046 (3)	0.3529 (3)	0.0491 (9)	
C29	−0.4845 (3)	0.3408 (3)	0.3635 (3)	0.0378 (8)	
C30	−0.5383 (3)	0.2232 (3)	0.3608 (2)	0.0371 (8)	
O21	−0.0582 (3)	0.7003 (3)	0.4724 (3)	0.0810 (10)	
H21B	−0.126 (3)	0.648 (3)	0.448 (3)	0.080*	
H21A	−0.022 (4)	0.698 (4)	0.5295 (16)	0.080*	
O22	−0.0730 (3)	0.2875 (2)	0.3291 (2)	0.0699 (8)	
O23	0.0758 (3)	0.7542 (2)	−0.0680 (2)	0.0539 (7)	
H23B	0.139 (3)	0.734 (4)	−0.063 (4)	0.080*	
H23A	0.091 (4)	0.809 (3)	−0.098 (3)	0.080*	

### Atomic displacement parameters (Å^2^)

**Table:**

	*U*^11^	*U*^22^	*U*^33^	*U*^12^	*U*^13^	*U*^23^
Ca1	0.0356 (4)	0.0336 (4)	0.0470 (4)	0.0079 (3)	0.0112 (3)	0.0073 (3)
S2	0.0317 (5)	0.0357 (5)	0.0573 (6)	0.0038 (4)	0.0169 (4)	0.0026 (4)
O1	0.0277 (12)	0.0306 (12)	0.0481 (14)	0.0090 (10)	0.0077 (10)	0.0067 (10)
O2	0.0272 (13)	0.0438 (14)	0.0663 (17)	0.0011 (11)	0.0060 (12)	0.0070 (12)
O3	0.0255 (13)	0.0534 (16)	0.083 (2)	0.0134 (12)	0.0080 (13)	0.0070 (15)
O4	0.0544 (17)	0.0368 (15)	0.104 (2)	0.0180 (13)	0.0249 (18)	0.0126 (15)
O5	0.0462 (16)	0.0341 (14)	0.112 (2)	0.0042 (12)	0.0365 (16)	−0.0017 (15)
O6	0.0365 (15)	0.0751 (19)	0.0498 (16)	0.0051 (14)	0.0038 (12)	0.0060 (14)
O7	0.0452 (15)	0.0417 (14)	0.0638 (17)	0.0107 (12)	0.0272 (13)	0.0079 (12)
O8	0.0415 (15)	0.0599 (18)	0.0611 (18)	0.0136 (13)	0.0134 (14)	0.0121 (15)
O9	0.056 (2)	0.0396 (16)	0.110 (3)	0.0005 (14)	0.0415 (18)	−0.0045 (16)
O10	0.075 (2)	0.0539 (18)	0.0548 (18)	−0.0136 (16)	−0.0018 (16)	0.0158 (16)
O11	0.0375 (15)	0.0555 (17)	0.0581 (18)	0.0041 (13)	0.0096 (13)	0.0097 (14)
O12	0.077 (2)	0.0357 (15)	0.071 (2)	0.0152 (15)	0.0338 (17)	0.0048 (15)
O13	0.069 (2)	0.079 (2)	0.071 (2)	0.0456 (17)	0.0370 (17)	0.0371 (18)
C1	0.043 (2)	0.038 (2)	0.045 (2)	0.0132 (16)	0.0130 (16)	0.0061 (16)
C2	0.058 (3)	0.039 (2)	0.058 (2)	0.0242 (19)	0.0158 (19)	0.0144 (17)
C3	0.051 (2)	0.058 (3)	0.059 (2)	0.031 (2)	0.0124 (19)	0.012 (2)
C4	0.032 (2)	0.058 (3)	0.067 (3)	0.0143 (18)	0.0086 (18)	0.007 (2)
C5	0.034 (2)	0.0360 (19)	0.057 (2)	0.0088 (16)	0.0074 (17)	0.0053 (16)
C6	0.0340 (18)	0.0350 (18)	0.0333 (17)	0.0119 (15)	0.0086 (14)	0.0039 (14)
C7	0.0328 (18)	0.0322 (17)	0.0322 (17)	0.0095 (14)	0.0073 (14)	0.0038 (13)
C8	0.0359 (19)	0.0288 (17)	0.048 (2)	0.0078 (15)	0.0110 (16)	0.0069 (15)
C9	0.0287 (18)	0.0381 (19)	0.0389 (19)	0.0031 (15)	0.0067 (14)	0.0057 (15)
C10	0.0298 (18)	0.0387 (19)	0.0399 (19)	0.0103 (15)	0.0076 (14)	0.0084 (15)
C11	0.0295 (18)	0.047 (2)	0.046 (2)	0.0126 (16)	0.0089 (15)	0.0082 (16)
C12	0.040 (2)	0.047 (2)	0.065 (3)	0.0236 (18)	0.0120 (18)	0.0141 (19)
C13	0.043 (2)	0.036 (2)	0.056 (2)	0.0152 (16)	0.0180 (17)	0.0113 (16)
C14	0.0336 (18)	0.0337 (18)	0.0451 (19)	0.0093 (15)	0.0133 (15)	0.0074 (15)
C15	0.0246 (16)	0.0385 (18)	0.0394 (18)	0.0108 (14)	0.0113 (14)	0.0084 (14)
S1	0.0318 (5)	0.0354 (5)	0.0513 (6)	0.0048 (4)	0.0067 (4)	0.0057 (4)
O14	0.0316 (13)	0.0314 (12)	0.0528 (14)	0.0078 (10)	0.0110 (11)	0.0072 (10)
O15	0.0353 (15)	0.0471 (15)	0.084 (2)	−0.0010 (12)	0.0207 (14)	0.0092 (14)
O16	0.0327 (15)	0.0594 (18)	0.115 (3)	0.0123 (13)	0.0231 (16)	0.0089 (19)
O17	0.0482 (17)	0.0341 (15)	0.133 (3)	0.0165 (13)	0.0234 (19)	0.0167 (16)
O18	0.0450 (16)	0.0344 (15)	0.117 (3)	0.0049 (13)	0.0109 (16)	0.0090 (15)
O19	0.0381 (14)	0.0573 (16)	0.0547 (16)	0.0056 (12)	0.0149 (12)	0.0051 (13)
O20	0.0424 (15)	0.0631 (17)	0.0486 (15)	0.0074 (13)	−0.0013 (12)	0.0090 (13)
C16	0.046 (2)	0.0358 (19)	0.056 (2)	0.0113 (17)	0.0063 (18)	0.0089 (17)
C17	0.045 (2)	0.053 (2)	0.075 (3)	0.018 (2)	0.000 (2)	0.007 (2)
C18	0.061 (3)	0.060 (3)	0.066 (3)	0.034 (2)	0.002 (2)	0.007 (2)
C19	0.075 (3)	0.039 (2)	0.072 (3)	0.023 (2)	0.014 (2)	0.014 (2)
C20	0.051 (2)	0.045 (2)	0.064 (3)	0.0120 (19)	0.015 (2)	0.0100 (19)
C21	0.043 (2)	0.0350 (19)	0.0396 (19)	0.0107 (16)	0.0095 (16)	0.0111 (15)
C22	0.042 (2)	0.0309 (18)	0.0414 (19)	0.0065 (16)	0.0105 (16)	0.0062 (15)
C23	0.045 (2)	0.0340 (19)	0.055 (2)	0.0047 (17)	0.0159 (18)	0.0086 (16)
C24	0.038 (2)	0.040 (2)	0.050 (2)	0.0037 (17)	0.0170 (17)	0.0067 (16)
C25	0.0315 (19)	0.0391 (19)	0.046 (2)	0.0062 (15)	0.0127 (15)	0.0041 (15)
C26	0.033 (2)	0.049 (2)	0.061 (2)	0.0096 (17)	0.0142 (17)	0.0027 (18)
C27	0.036 (2)	0.047 (2)	0.082 (3)	0.0186 (18)	0.0180 (19)	0.007 (2)
C28	0.042 (2)	0.040 (2)	0.066 (3)	0.0147 (17)	0.0131 (18)	0.0093 (18)
C29	0.0322 (18)	0.0332 (18)	0.046 (2)	0.0076 (15)	0.0108 (15)	0.0045 (15)
C30	0.0335 (18)	0.0367 (19)	0.0392 (19)	0.0092 (15)	0.0087 (15)	0.0054 (15)
O21	0.065 (2)	0.079 (2)	0.077 (2)	−0.0047 (18)	0.0171 (19)	0.003 (2)
O22	0.072 (2)	0.0505 (17)	0.087 (2)	0.0173 (15)	0.0254 (17)	0.0091 (15)
O23	0.0456 (16)	0.0439 (16)	0.0679 (18)	0.0051 (13)	0.0177 (14)	0.0126 (13)

### Geometric parameters (Å, °)

**Table:**

Ca1—O12	2.317 (3)	C9—C10	1.432 (5)
Ca1—O9	2.331 (3)	C10—C15	1.389 (4)
Ca1—O10	2.383 (3)	C10—C11	1.417 (5)
Ca1—O13	2.383 (3)	C11—C12	1.359 (5)
Ca1—O11	2.453 (3)	C12—C13	1.391 (5)
Ca1—O8	2.456 (3)	C12—H12	0.9300
Ca1—O7	2.481 (3)	C13—C14	1.409 (5)
Ca1—H8B	2.74 (4)	C14—C15	1.393 (4)
Ca1—H9B	2.77 (4)	S1—O19	1.441 (3)
S2—O7	1.445 (3)	S1—O20	1.448 (3)
S2—O6	1.446 (3)	S1—O18	1.456 (3)
S2—O5	1.464 (3)	S1—C29	1.769 (3)
S2—C14	1.768 (3)	O14—C22	1.363 (4)
O1—C7	1.359 (4)	O14—C30	1.368 (4)
O1—C15	1.368 (4)	O15—C24	1.251 (4)
O2—C9	1.267 (4)	O16—C26	1.344 (4)
O3—C11	1.351 (4)	O16—H16	0.8200
O3—H3	0.8200	O17—C28	1.345 (4)
O4—C13	1.340 (4)	O17—H17	0.8200
O4—H4	0.8200	C16—C17	1.375 (5)
O8—H8A	0.82 (3)	C16—C21	1.392 (5)
O8—H8B	0.82 (4)	C16—H16A	0.9300
O9—H9A	0.83 (3)	C17—C18	1.376 (6)
O9—H9B	0.82 (3)	C17—H17A	0.9300
O10—H10A	0.82 (3)	C18—C19	1.372 (6)
O10—H10B	0.82 (3)	C18—H18	0.9300
O11—H11B	0.82 (3)	C19—C20	1.378 (6)
O11—H11A	0.82 (4)	C19—H19	0.9300
O12—H12A	0.82 (4)	C20—C21	1.377 (5)
O12—H12B	0.81 (3)	C20—H20	0.9300
O13—H13B	0.82 (3)	C21—C22	1.474 (5)
O13—H13A	0.82 (3)	C22—C23	1.340 (5)
C1—C2	1.381 (5)	C23—C24	1.435 (5)
C1—C6	1.403 (4)	C23—H23	0.9300
C1—H1	0.9300	C24—C25	1.446 (5)
C2—C3	1.372 (5)	C25—C30	1.387 (5)
C2—H2	0.9300	C25—C26	1.413 (5)
C3—C4	1.381 (5)	C26—C27	1.363 (5)
C3—H3A	0.9300	C27—C28	1.393 (5)
C4—C5	1.380 (5)	C27—H27	0.9300
C4—H4A	0.9300	C28—C29	1.412 (5)
C5—C6	1.391 (5)	C29—C30	1.395 (4)
C5—H5	0.9300	O21—H21B	0.82 (3)
C6—C7	1.468 (4)	O21—H21A	0.83 (4)
C7—C8	1.354 (4)	O23—H23B	0.82 (4)
C8—C9	1.429 (5)	O23—H23A	0.82 (4)
C8—H8	0.9300		
			
O12—Ca1—O9	166.74 (12)	C8—C7—C6	126.7 (3)
O12—Ca1—O10	79.54 (12)	O1—C7—C6	111.8 (3)
O9—Ca1—O10	87.19 (13)	C7—C8—C9	120.9 (3)
O12—Ca1—O13	78.35 (12)	C7—C8—H8	119.6
O9—Ca1—O13	113.01 (13)	C9—C8—H8	119.6
O10—Ca1—O13	142.40 (12)	O2—C9—C8	121.9 (3)
O12—Ca1—O11	95.00 (11)	O2—C9—C10	121.5 (3)
O9—Ca1—O11	81.03 (11)	C8—C9—C10	116.5 (3)
O10—Ca1—O11	72.82 (10)	C15—C10—C11	117.6 (3)
O13—Ca1—O11	79.24 (11)	C15—C10—C9	120.0 (3)
O12—Ca1—O8	82.43 (11)	C11—C10—C9	122.4 (3)
O9—Ca1—O8	94.11 (11)	O3—C11—C12	119.7 (3)
O10—Ca1—O8	74.99 (11)	O3—C11—C10	119.6 (3)
O13—Ca1—O8	130.90 (11)	C12—C11—C10	120.7 (3)
O11—Ca1—O8	147.62 (10)	C11—C12—C13	120.7 (3)
O12—Ca1—O7	113.40 (10)	C11—C12—H12	119.6
O9—Ca1—O7	77.30 (9)	C13—C12—H12	119.6
O10—Ca1—O7	142.23 (12)	O4—C13—C12	115.9 (3)
O13—Ca1—O7	75.02 (10)	O4—C13—C14	123.2 (3)
O11—Ca1—O7	136.16 (10)	C12—C13—C14	120.8 (3)
O8—Ca1—O7	72.10 (10)	C15—C14—C13	117.0 (3)
O12—Ca1—H8B	99.3 (5)	C15—C14—S2	120.0 (2)
O9—Ca1—H8B	77.3 (5)	C13—C14—S2	122.9 (3)
O10—Ca1—H8B	76.7 (10)	O1—C15—C10	120.3 (3)
O13—Ca1—H8B	136.9 (10)	O1—C15—C14	116.6 (3)
O11—Ca1—H8B	143.1 (10)	C10—C15—C14	123.1 (3)
O8—Ca1—H8B	17.0 (5)	O19—S1—O20	111.90 (16)
O7—Ca1—H8B	66.5 (10)	O19—S1—O18	112.13 (18)
O12—Ca1—H9B	169.3 (9)	O20—S1—O18	112.59 (17)
O9—Ca1—H9B	15.9 (7)	O19—S1—C29	107.48 (15)
O10—Ca1—H9B	98.9 (8)	O20—S1—C29	107.24 (16)
O13—Ca1—H9B	108.1 (10)	O18—S1—C29	105.01 (16)
O11—Ca1—H9B	94.7 (8)	C22—O14—C30	120.7 (3)
O8—Ca1—H9B	86.9 (9)	C26—O16—H16	109.5
O7—Ca1—H9B	61.6 (7)	C28—O17—H17	109.5
H8B—Ca1—H9B	70.1 (11)	C17—C16—C21	120.1 (3)
O7—S2—O6	112.63 (17)	C17—C16—H16A	120.0
O7—S2—O5	112.18 (16)	C21—C16—H16A	120.0
O6—S2—O5	111.20 (18)	C16—C17—C18	120.5 (4)
O7—S2—C14	108.38 (16)	C16—C17—H17A	119.7
O6—S2—C14	106.78 (16)	C18—C17—H17A	119.7
O5—S2—C14	105.20 (16)	C19—C18—C17	119.6 (4)
C7—O1—C15	120.8 (2)	C19—C18—H18	120.2
C11—O3—H3	109.5	C17—C18—H18	120.2
C13—O4—H4	109.5	C18—C19—C20	120.3 (4)
S2—O7—Ca1	141.98 (14)	C18—C19—H19	119.9
Ca1—O8—H8A	106 (3)	C20—C19—H19	119.9
Ca1—O8—H8B	102 (3)	C21—C20—C19	120.6 (4)
H8A—O8—H8B	114 (5)	C21—C20—H20	119.7
Ca1—O9—H9A	129 (3)	C19—C20—H20	119.7
Ca1—O9—H9B	114 (3)	C20—C21—C16	118.9 (3)
H9A—O9—H9B	114 (5)	C20—C21—C22	120.7 (3)
Ca1—O10—H10A	117 (3)	C16—C21—C22	120.4 (3)
Ca1—O10—H10B	127 (3)	C23—C22—O14	121.4 (3)
H10A—O10—H10B	112 (5)	C23—C22—C21	126.8 (3)
Ca1—O11—H11B	125 (3)	O14—C22—C21	111.8 (3)
Ca1—O11—H11A	128 (3)	C22—C23—C24	121.8 (3)
H11B—O11—H11A	104 (5)	C22—C23—H23	119.1
Ca1—O12—H12A	121 (3)	C24—C23—H23	119.1
Ca1—O12—H12B	134 (4)	O15—C24—C23	123.3 (3)
H12A—O12—H12B	105 (5)	O15—C24—C25	121.3 (3)
Ca1—O13—H13B	122 (3)	C23—C24—C25	115.4 (3)
Ca1—O13—H13A	129 (4)	C30—C25—C26	118.1 (3)
H13B—O13—H13A	109 (5)	C30—C25—C24	120.1 (3)
C2—C1—C6	119.9 (3)	C26—C25—C24	121.8 (3)
C2—C1—H1	120.1	O16—C26—C27	119.8 (3)
C6—C1—H1	120.1	O16—C26—C25	120.0 (3)
C3—C2—C1	120.7 (3)	C27—C26—C25	120.3 (3)
C3—C2—H2	119.6	C26—C27—C28	120.7 (3)
C1—C2—H2	119.6	C26—C27—H27	119.6
C2—C3—C4	119.9 (4)	C28—C27—H27	119.6
C2—C3—H3A	120.0	O17—C28—C27	116.5 (3)
C4—C3—H3A	120.0	O17—C28—C29	122.3 (3)
C5—C4—C3	120.3 (4)	C27—C28—C29	121.2 (3)
C5—C4—H4A	119.9	C30—C29—C28	116.4 (3)
C3—C4—H4A	119.9	C30—C29—S1	120.7 (3)
C4—C5—C6	120.4 (3)	C28—C29—S1	122.8 (3)
C4—C5—H5	119.8	O14—C30—C25	120.5 (3)
C6—C5—H5	119.8	O14—C30—C29	116.2 (3)
C5—C6—C1	118.8 (3)	C25—C30—C29	123.3 (3)
C5—C6—C7	120.7 (3)	H21B—O21—H21A	115 (5)
C1—C6—C7	120.4 (3)	H23B—O23—H23A	103 (5)
C8—C7—O1	121.4 (3)		
			
O6—S2—O7—Ca1	102.4 (3)	C13—C14—C15—O1	−178.3 (3)
O5—S2—O7—Ca1	−23.9 (3)	S2—C14—C15—O1	4.8 (4)
C14—S2—O7—Ca1	−139.7 (2)	C13—C14—C15—C10	2.8 (5)
O12—Ca1—O7—S2	−176.2 (2)	S2—C14—C15—C10	−174.1 (3)
O9—Ca1—O7—S2	12.1 (3)	C21—C16—C17—C18	−0.9 (6)
O10—Ca1—O7—S2	80.2 (3)	C16—C17—C18—C19	−0.3 (7)
O13—Ca1—O7—S2	−106.3 (3)	C17—C18—C19—C20	1.5 (7)
O11—Ca1—O7—S2	−50.2 (3)	C18—C19—C20—C21	−1.4 (7)
O8—Ca1—O7—S2	110.7 (3)	C19—C20—C21—C16	0.2 (6)
C6—C1—C2—C3	0.1 (5)	C19—C20—C21—C22	−179.4 (4)
C1—C2—C3—C4	0.6 (6)	C17—C16—C21—C20	1.0 (6)
C2—C3—C4—C5	−0.6 (6)	C17—C16—C21—C22	−179.4 (4)
C3—C4—C5—C6	−0.1 (6)	C30—O14—C22—C23	1.6 (5)
C4—C5—C6—C1	0.7 (5)	C30—O14—C22—C21	−178.6 (3)
C4—C5—C6—C7	179.9 (3)	C20—C21—C22—C23	6.8 (6)
C2—C1—C6—C5	−0.8 (5)	C16—C21—C22—C23	−172.7 (4)
C2—C1—C6—C7	−179.9 (3)	C20—C21—C22—O14	−173.0 (3)
C15—O1—C7—C8	1.3 (5)	C16—C21—C22—O14	7.4 (5)
C15—O1—C7—C6	−178.5 (3)	O14—C22—C23—C24	−0.6 (5)
C5—C6—C7—C8	−174.4 (3)	C21—C22—C23—C24	179.6 (3)
C1—C6—C7—C8	4.7 (5)	C22—C23—C24—O15	179.0 (3)
C5—C6—C7—O1	5.4 (4)	C22—C23—C24—C25	−1.2 (5)
C1—C6—C7—O1	−175.5 (3)	O15—C24—C25—C30	−178.0 (3)
O1—C7—C8—C9	−1.0 (5)	C23—C24—C25—C30	2.1 (5)
C6—C7—C8—C9	178.8 (3)	O15—C24—C25—C26	1.0 (6)
C7—C8—C9—O2	178.9 (3)	C23—C24—C25—C26	−178.9 (3)
C7—C8—C9—C10	−0.4 (5)	C30—C25—C26—O16	178.1 (3)
O2—C9—C10—C15	−177.9 (3)	C24—C25—C26—O16	−1.0 (6)
C8—C9—C10—C15	1.3 (5)	C30—C25—C26—C27	−0.8 (6)
O2—C9—C10—C11	1.8 (5)	C24—C25—C26—C27	−179.9 (4)
C8—C9—C10—C11	−178.9 (3)	O16—C26—C27—C28	−177.2 (4)
C15—C10—C11—O3	178.8 (3)	C25—C26—C27—C28	1.6 (6)
C9—C10—C11—O3	−0.9 (5)	C26—C27—C28—O17	177.9 (4)
C15—C10—C11—C12	−0.5 (5)	C26—C27—C28—C29	−1.4 (6)
C9—C10—C11—C12	179.8 (3)	O17—C28—C29—C30	−178.9 (4)
O3—C11—C12—C13	−177.4 (3)	C27—C28—C29—C30	0.3 (6)
C10—C11—C12—C13	1.9 (6)	O17—C28—C29—S1	−1.4 (6)
C11—C12—C13—O4	178.1 (4)	C27—C28—C29—S1	177.9 (3)
C11—C12—C13—C14	−1.0 (6)	O19—S1—C29—C30	63.6 (3)
O4—C13—C14—C15	179.7 (3)	O20—S1—C29—C30	−56.9 (3)
C12—C13—C14—C15	−1.3 (5)	O18—S1—C29—C30	−176.8 (3)
O4—C13—C14—S2	−3.5 (5)	O19—S1—C29—C28	−113.8 (3)
C12—C13—C14—S2	175.5 (3)	O20—S1—C29—C28	125.7 (3)
O7—S2—C14—C15	−63.6 (3)	O18—S1—C29—C28	5.7 (4)
O6—S2—C14—C15	58.0 (3)	C22—O14—C30—C25	−0.6 (5)
O5—S2—C14—C15	176.3 (3)	C22—O14—C30—C29	179.4 (3)
O7—S2—C14—C13	119.7 (3)	C26—C25—C30—O14	179.6 (3)
O6—S2—C14—C13	−118.7 (3)	C24—C25—C30—O14	−1.2 (5)
O5—S2—C14—C13	−0.5 (4)	C26—C25—C30—C29	−0.3 (5)
C7—O1—C15—C10	−0.3 (4)	C24—C25—C30—C29	178.8 (3)
C7—O1—C15—C14	−179.2 (3)	C28—C29—C30—O14	−179.4 (3)
C11—C10—C15—O1	179.2 (3)	S1—C29—C30—O14	3.0 (4)
C9—C10—C15—O1	−1.0 (5)	C28—C29—C30—C25	0.5 (5)
C11—C10—C15—C14	−2.0 (5)	S1—C29—C30—C25	−177.1 (3)
C9—C10—C15—C14	177.8 (3)		

### Hydrogen-bond geometry (Å, °)

**Table:**

*D*—H···*A*	*D*—H	H···*A*	*D*···*A*	*D*—H···*A*
O3—H3···O2	0.82	1.85	2.576 (4)	148
O4—H4···O5	0.82	1.82	2.579 (4)	153
O8—H8B···O17^i^	0.82 (4)	2.20 (3)	2.934 (4)	149 (4)
O8—H8A···O20^ii^	0.82 (3)	1.97 (3)	2.790 (4)	176 (5)
O9—H9B···O5	0.82 (4)	1.99 (4)	2.780 (4)	163 (5)
O9—H9A···O23^iii^	0.83 (3)	1.92 (3)	2.743 (4)	175 (5)
O10—H10B···O20^ii^	0.82 (3)	2.13 (2)	2.874 (4)	151 (5)
O10—H10A···O22	0.82 (3)	1.90 (3)	2.715 (4)	171 (5)
O11—H11B···O6^iii^	0.82 (3)	2.36 (3)	3.024 (4)	139 (4)
O11—H11A···O19	0.82 (4)	2.19 (4)	3.003 (4)	175 (5)
O12—H12B···O15^iv^	0.81 (3)	1.98 (3)	2.771 (4)	165 (5)
O12—H12A···O21	0.82 (4)	1.88 (4)	2.695 (5)	177 (4)
O13—H13B···O3^v^	0.82 (4)	2.05 (4)	2.867 (4)	174 (5)
O13—H13A···O23	0.82 (3)	2.00 (3)	2.819 (4)	173 (5)
O16—H16···O15	0.82	1.83	2.567 (4)	148
O17—H17···O18	0.82	1.80	2.556 (4)	153
O21—H21B···O18	0.82 (3)	2.05 (4)	2.870 (4)	176 (5)
O21—H21A···O22^ii^	0.82 (3)	2.01 (3)	2.826 (5)	173 (5)
O23—H23A···O2^vi^	0.82 (4)	2.00 (4)	2.816 (4)	171 (5)
O23—H23B···O6	0.82 (4)	1.95 (4)	2.755 (4)	168 (5)
C12—H12···O11^i^	0.93	2.55	3.462 (5)	168
C20—H20···O8^vii^	0.93	2.52	3.410 (5)	161

Symmetry codes: (i) *x*+1, *y*, *z*; (ii) −*x*, −*y*+1, −*z*+1; (iii) −*x*, −*y*+1, −*z*; (iv) *x*+1, *y*+1, *z*; (v) *x*−1, *y*, *z*; (vi) −*x*+1, −*y*+2, −*z*; (vii) *x*−1, *y*−1, *z*.
